# Mesenchymal Stem Cells Immunosuppressed IL-22 in Patients with Immune Thrombocytopenia via Soluble Cellular Factors

**DOI:** 10.1155/2015/316351

**Published:** 2015-10-04

**Authors:** Mei Wu, Hongfeng Ge, Shue Li, Hailiang Chu, Shili Yang, Xiaoxing Sun, Zhenxia Zhou, Xiongpeng Zhu

**Affiliations:** ^1^Department of Hematology, The People's Hospital of Bozhou, Bozhou 236800, China; ^2^Department of Hematology, First Hospital of Quanzhou Affiliated to Fujian Medical University, Quanzhou 362000, China

## Abstract

Mesenchymal stem cells are immunoregulation cells. IL-22 plays an important role in the pathogenesis of immune thrombocytopenia. However, the effects of mesenchymal stem cells on IL-22 production in patients with immune thrombocytopenia remain unclear. Flow cytometry analyzed immunophenotypes of mesenchymal stem cells; differentiation of mesenchymal stem cells was observed by oil red O and Alizarin red S staining; cell proliferation suppression was measured with MTS; IL-22 levels of cell-free supernatants were determined by ELISA. Mesenchymal stem cells inhibited the proliferation of activated CD4^+^T cells; moreover, mesenchymal stem cells immunosuppressed IL-22 by soluble cellular factors but not PGE2. These results suggest that mesenchymal stem cells may be a therapeutic strategy for patients with immune thrombocytopenia.

## 1. Introduction

Mesenchymal stem cells (MSCs) are multipotent cells and are able to differentiate into mature mesenchymal cells such as osteoblasts, adipocytes, and chondroblasts [1]. MSCs can be obtained from many tissues including adult bone marrow (BM), adipose tissue (AD), muscle, term placental chorionic villi (CV), cord blood, and umbilical cord (UC) [2–7]. But UC-MSCs are a more promising source [8]. Due to their stronger immunoregulation, MSCs have been widely applied for treatment of all kinds of diseases, for example, graft-versus-host disease (GVHD) [9], experimental autoimmune encephalomyelitis (EAE) [10], Crohn's disease (CD) [11], and rheumatoid arthritis (RA) [12].

Immune thrombocytopenia (ITP), also known as idiopathic thrombocytopenic purpura, is an autoimmune disease. Because of antiplatelet autoantibodies in patients, platelets are destroyed in large numbers and platelet count is lower obviously in peripheral blood. The etiology of ITP is not clear. Therefore, the diagnosis of ITP is exclusive, and there are no specific or sensitive laboratory methods used to detect these antibodies in clinic [13]. It is known that T cells abnormalities play an important role in the pathogenesis of ITP. T cells related cytokine abnormalities are one of the T cells abnormalities [14, 15]. Many studies found that the concentration of IL-22 produced by T cell subsets increased significantly in ITP patients [16–18]. However, the effect of UC-MSCs on ITP patients remains unclear.

In the present study, our data suggest that UC-MSCs inhibited the proliferation of CD4^+^T cells and immunosuppressed the production of IL-22 in ITP patients through soluble cellular factors.

## 2. Materials and Methods

### 2.1. The Isolation of UC-MSCs

Umbilical cords were obtained from our hospital's obstetrical department with informed consent. Human tissue collection for research was approved by the Medical Ethics Committee of Anhui Province in China. Isolation of human umbilical cord mesenchymal stem cells (UC-MSCs) was performed as described [7].

### 2.2. Immunophenotype Analysis by Flow Cytometry

UC-MSCs were stained with PE-conjugated antibody against CD11b, CD29, CD44, CD45, CD54, CD73, CD80, CD86, CD90, CD105, CD106, HLA-DR, nestin, and sox-2 or FITC-conjugated antibody against CD19, CD31, CD34, and HLA-ABC. The IgG1-PE and IgG1-FITC were used as negative controls. Antibodies (BD Pharmingen) were used according to manufacturer's protocol and were analyzed by flow cytometry.

### 2.3. The Differentiation and Staining Assays of UC-MSCs

2 × 10^4^ UC-MSCs were cultured by DMEM/F12 media containing 10% fetal bovine serum (FBS) in 24-well plates for 24 hours. Then, the media were changed with osteogenic or adipogenic induction media for 3 weeks; cells were observed by Alizarin red S or oil red O solution, respectively.

### 2.4. Preparation of Human CD4^+^T Cells

Human mononuclear cells from patients with ITP were isolated by Ficoll-Paque (Axis-Shield). Then, CD4^+^T cells were obtained with magnetic MicroBead kits (Miltenyi Biotec). The purity of CD4^+^T cells was more than 95% (data not shown).

### 2.5. Coculture Experiment of UC-MSCs and CD4^+^T Cells

UC-MSCs irradiated by 30 Gy were preplated and were allowed to adhere for 5 h at 37°C; CD4^+^T cells were added at a ratio of 1 : 10 for 72 h.

### 2.6. Cell Proliferation Assay

Cell proliferation was measured with an MTS kit (Promega) according to manufacturer's protocol. Absorbance was detected at 490 nm on BioTek reader (BIO-RAD).

### 2.7. Total RNA Extraction, Reverse Transcription, and Real-Time PCR Analysis

CD4^+^T cell was collected. RNA of CD4^+^T cell was extracted with E.Z.N.A. Total RNA Kit I (OMEGA). cDNA synthesis was done with the MLV RT kit (Invitrogen). Polymerase chain reaction analyses were performed by Platinum SYBR Green qPCR SuperMix-UDG w/ROX on an Applied Biosystems 7300 Real-Time PCR System. The IL-22 mRNA was expressed with ΔΔCt values. The primer of human IL-22 is 5′-ACAACACAGACGTTCGTCTCATTG-3′ and 5′-GAA CAGCACTTCTTCAAGGGTGA-3′.

### 2.8. Enzyme-Linked Immunosorbent Assay (ELISA) Measured IL-22 Concentration

IL-22 concentration of cell-free supernatants was tested by Human IL-22 ELISA assay kits (Peprotech) according to manufacturer's protocol.

### 2.9. Statistical Analysis

The SPSS 17.0 software package analyzed data. Data are presented as mean ± SD. Comparisons were performed by one-way ANOVA. *P* < 0.05 was considered significant.

## 3. Results

### 3.1. The Characteristics of UC-MSCs

**Figure 1:**
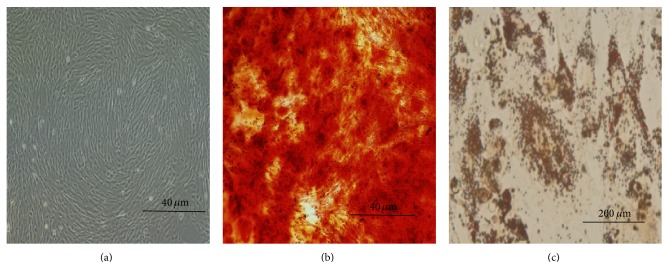
The characteristics of UC-MSCs. (a) Morphology of UC-MSCs; (b) the osteogenic differentiation of UC-MSCs; (c) adipogenic differentiation of UC-MSCs.

**Figure 2:**
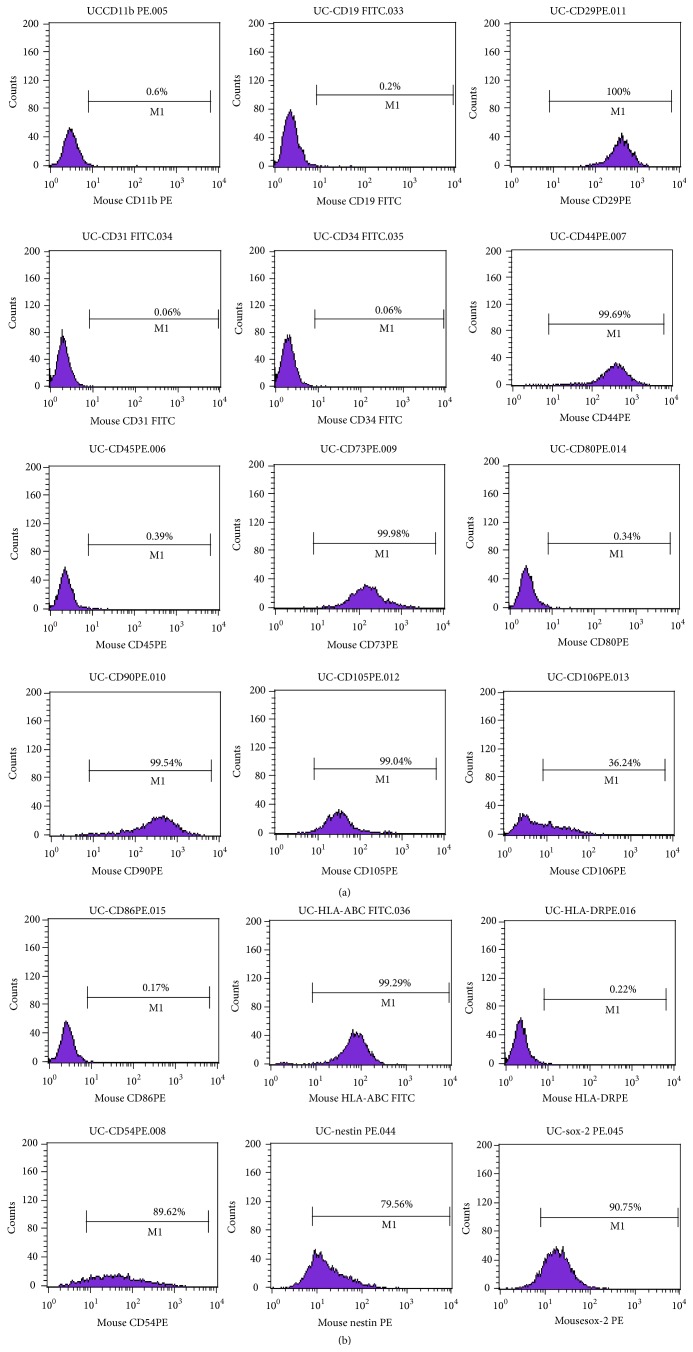
Immunophenotypes of UC-MSCs. UC-MSCs expressed CD29, CD44, CD54, CD73, CD90, CD105, CD106, HLA-ABC, nestin, and sox-2 but did not express CD11b, CD14, CD19, CD34, CD45, CD80, CD86, and HLA-DR. This figure shows one of the three independent experiments' results.

As shown in Figure 1(a), UC-MSCs isolated from umbilical cord were fibroblast-like cells. They were induced successfully into osteoblasts and adipocytes observed by Alizarin red S and oil red O staining in specific medias (Figures 1(b) and 1(c)). Furthermore, flow cytometry showed that UC-MSCs were positive for CD29, CD44, CD54, CD73, CD90, CD105, CD106, HLA-ABC, nestin, and sox-2 and negative for CD11b, CD31, CD19, CD34, CD45, CD80, CD86, and HLA-DR (Figure 2, Table 1).

### 3.2. UC-MSCs Suppressed the Proliferation of CD4^+^T Cells

**Figure 3:**
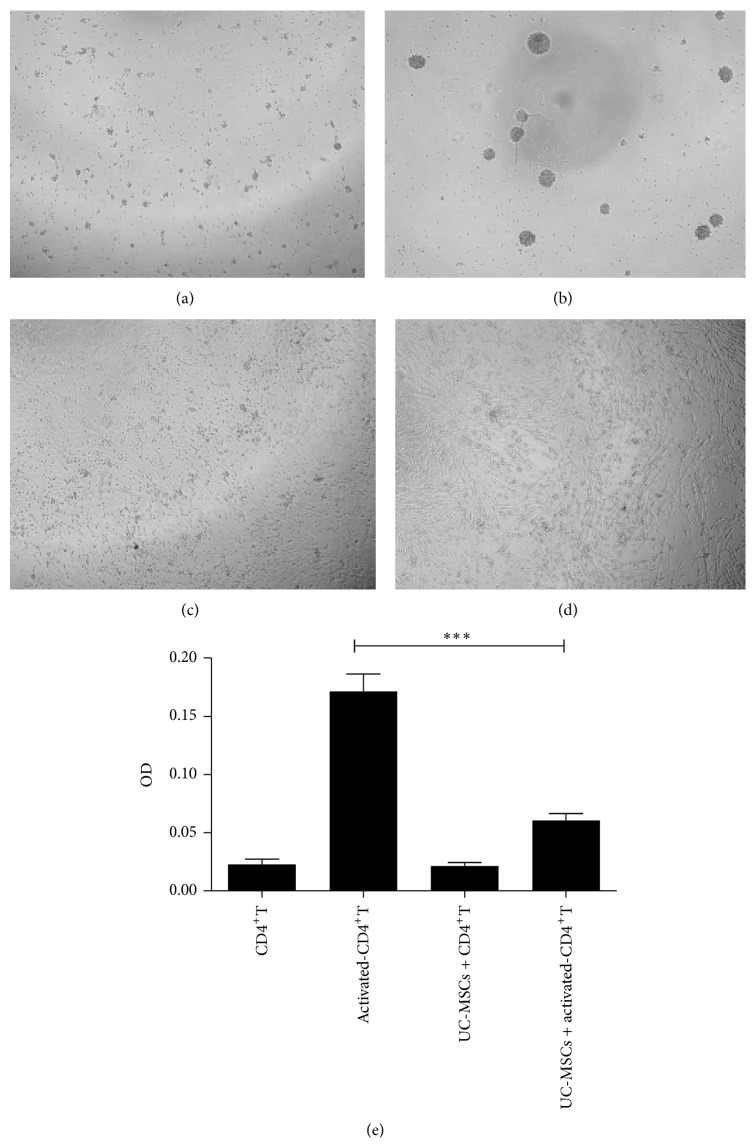
UC-MSCs suppressed proliferation of CD4^+^T cells. (a) Nonactivated CD4^+^T cells; (b) activated CD4^+^T cells; (c) cocultured nonactivated CD4^+^T cells with UC-MSCs; (d) cocultured activated CD4^+^T cells with UC-MSCs; magnification: 40x; (e) proliferation was evaluated by MTS. Data represent one of the three independent experiments, each performed in triplicate. ^*∗∗∗*^ *P* < 0.001.

To examine the effect of UC-MSCs on CD4^+^T cells from ITP patients, we treated CD4^+^T cells with UC-MSCs in the absence or presence of stimuli (CD3/CD28) and found that CD4^+^T cells could not proliferate culturing with UC-MSCs or in the absence of stimuli (Figures 3(a) and 3(c)). However, CD4^+^T cells appeared to cluster in the presence of stimuli (Figure 3(b)). Most important, when cocultured with UC-MSCs, activated CD4^+^T cells grew in a spreading pattern (Figure 3(d)). MTS was used to evaluate further the proliferation of CD4^+^T cells (Figure 3(e)); the result of MTS was consistent with the proliferation of CD4^+^T cells alone or culture with UC-MSC in the absence or presence of stimuli.

### 3.3. UC-MSCs Inhibited CD4^+^T Cells Secreting IL-22

**Figure 4:**
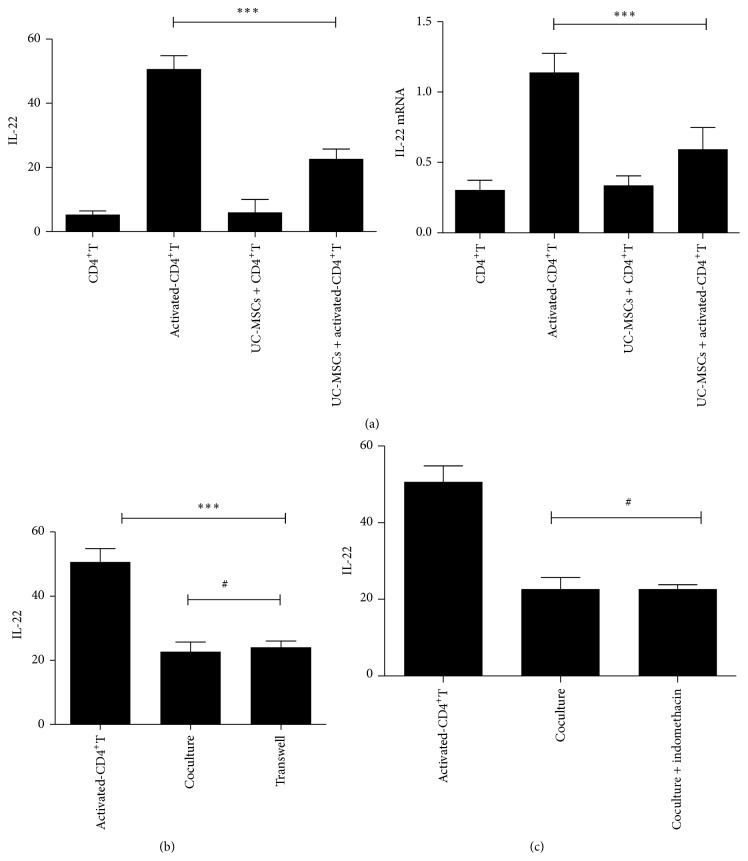
UC-MSCs immunosuppressed IL-22 by soluble cellular factors but not PGE2. (a) UC-MSCs inhibited CD4^+^T cells secreting IL-22 and the expression of IL-22 mRNA; (b) the immunoregulation of UC-MSCs on IL-22 was mediated by soluble cellular factors; (c) PGE2 did not involve in the immunoregulation of UC-MSCs on IL-22. Data represent one of the three independent experiments, each performed in triplicate. ^*∗∗∗*^ *P* < 0.001 and ^#^ *P* > 0.05.

We measured the production and mRNA of IL-22 to investigate the immunomodulation of UC-MSCs on IL-22. As shown in Figure 4(a), nonactivated CD4^+^T cells or CD4^+^T cells cocultured with UC-MSCs without stimuli produced low level of IL-22. But the concentration of IL-22 was increased enormously in activated CD4^+^T cells. When activated CD4^+^T cells were cocultured with UC-MSCs, higher levels of IL-22 were reduced again (*P* < 0.001). Furthermore, this phenomenon was also observed in expression of IL-22 mRNA. Thus, UC-MSCs had strong immunosuppression in CD4^+^T cells secreting IL-22.

### 3.4. UC-MSCs Immunomodulated IL-22 by Soluble Cellular Factors

It is known that MSCs play their immunosuppressive effects by cell-cell contact or soluble cellular factors. To examine it, we performed coculture experiments using the Transwell system. Transwell physically separated CD4^+^T cells from UC-MSCs; it only allows for soluble cellular factors to permeate. We found that UC-MSCs were also able to inhibit dramatically the secretion of IL-22 without cell-cell contact (*P* < 0.001). Furthermore, the degree of IL-22 inhibition by UC-MSCs in coculture separated by Transwell was not different significantly from those in coculture which was cell-cell contact (*P* > 0.05), indicating that the immunoregulation of UC-MSCs on IL-22 was mediated by soluble cellular factors (Figure 4(b)).

### 3.5. PGE2 Did Not Mediate the Immunomodulation of UC-MSCs on IL-22

Prostaglandin E2 (PGE2) is one of the soluble cellular factors mediating the immunoregulation of MSCs. Therefore, we performed coculture experiments with indometacin (10 mM), the inhibitors of PGE2. We found that the level of IL-22 decreased by UC-MSCs was not improved in coculture with indometacin compared to group without indometacin (*P* > 0.05). Together, this data suggests that UC-MSCs immunoregulated IL-22 via soluble cellular factors but not PGE2 (Figure 4(c)).

## 4. Discussion

In this present study, we have successfully demonstrated a previously uncharacterized fact that UC-MSCs possessed strong immunosuppressive capacity on IL-22 in patients with ITP.

Immunoregulation is one of the biological characteristics of MSCs; they can modulate the function of different immune cells such as T cells, B cells, neutrophils, natural killer (NK) cells, and dendritic cells (DC) [19–23]. MSCs block the division of T cells, preventing irreversible G0/G1 phase arrest and reducing the secretion of proinflammatory cytokines such as IFN-*γ* and TNF-*α* [24]. The immunomodulatory activity of the MSCs is also exerted through the inhibition of DC differentiation and maturation of antigen-presenting cells [25]. In addition, we had proved in our previous study that UC-MSCs suppressed significantly the mRNA expression of TNF-*α*, IL-21, IL-22, and IL-26 in CD4^+^T cells [8].

To our knowledge, IL-22, a newly defined cytokine, is one member of the IL-10 cytokine family, produced by CD4 cells, Th22 cells, Th17 cells, and NK cells. IL-22 can combine with its counterpart receptor complex which is composed of the IL-22R1 and IL-10R2, and its signal intracellularly is mediated by transcription factor JAK/STAT [26]. In different circumstance, IL-22 may play a protective or a pathogenic role. For instance, Liang and colleagues reported that IL-22 inhibited the development of bleomycin-induced pulmonary fibrosis [27]. However, IL-22 plays a pathogenic role in ITP [18]. The effects of MSCs on IL-22 in patients with ITP are unclear. In this study, we found that UC-MSCs had ability to immunoregulate IL-22 in patients with ITP. They decreased the IL-22 level of cell-free supernatants* in vitro*. In general, MSCs exert immunomodulatory effects through cell-cell contact or soluble cellular factors. We also found that UC-MSCs downregulated IL-22 when UC-MSCs were separated from CD4^+^T cells by Transwell. We come to conclusion that UC-MSCs suppressed the secretion of IL-22 by soluble cellular factors. Soluble cellular factors include NO, TGF-*β*1, PGE2, IDO1, HGF, IL-6, IL-10, and HLA-G [11, 28–34]. PGE2 is derived from the cyclooxygenase metabolism of arachidonic acid and is generated in large quantities by both macrophages and neighboring epithelial cells [35, 36]. PGE2 as an important regulator of the immune response shifts the balance towards a T helper type 2 response and promotes memory cell formation [37]. Our colleagues demonstrated that PGE2 is involved in the immunoregulation effect of MSCs [29, 38]. However, we added indomethacin which is the blocker of PGE2 into the group of UC-MSCs cocultured with CD4^+^T cells and found that indomethacin did not reverse the immunosuppressive effect of UC-MSCs on IL-22.

In summary, this study reports for the first time that UC-MSCs downregulate IL-22 of ITP patients through soluble cellular factors but not PGE2.

## Figures and Tables

**Table 1:** Immunophenotypes of UC-MSCs.

Surface marker	Positive rate
CD11b	−
CD31	−
CD73	**++++**
CD90	**++++**
CD80	−
CD106	**++**
HLA-ABC	**++++**
HLA-DR	−
Sox-2	**++++**
CD19	−
CD44	**++++**
CD34	−
CD54	**++++**
CD45	−
CD105	**++++**
CD86	−
Nestin	**++++**

− negative, +~++++ positive, + 1–25%, ++ 25–50%, +++ 50–75%, and ++++ >75%.
